# Successful treatment of cardiac sarcoidosis based on clinical suspicion and advanced cardiac imaging: A case report

**DOI:** 10.1097/MD.0000000000027814

**Published:** 2022-08-26

**Authors:** Jose S. Aguilar-Gallardo, Javier Arreaza, Alaa Omar, Glenmore Lasam, Johanna P. Contreras

**Affiliations:** a Department of Medicine, Mount Sinai Morningside, Icahn School of Medicine at Mount Sinai, New York, NY; b Division of Cardiology, Mount Sinai Morningside, Icahn School of Medicine at Mount Sinai, New York, NY; c Zena and Michael A. Wiener Cardiovascular Institute, Mount Sinai Hospital, Icahn School of Medicine at Mount Sinai, New York, NY.

**Keywords:** advance cardiac imaging, cardiac FDG-PET, cardiac magnetic resonance, cardiac sarcoidosis, endomyocardial biopsy

## Abstract

Confirming the diagnosis of cardiac sarcoidosis (CS) is a challenging task as we often do not count with histopathologic evidence. However, prompt initiation of treatment is sometimes necessary, and advanced cardiac imaging along with key clinical findings can play a crucial role in the diagnostic workup.

**Patient concerns:**

A 77-year-old male with a history of heart failure presented with chest pain and shortness of breath. He was found to have an acute drop in left ventricular ejection fraction associated with frequent premature ventricular contractions and nonsustained ventricular tachycardia. Coronary angiogram was negative for acute coronary syndrome. Advanced cardiac imaging with cardiac magnetic resonance raised suspicion of CS, and steroids were started empirically. Endomyocardial biopsy was attempted but was not successful.

**Diagnosis:**

The patient’s presentation was highly suggestive of cardiac sarcoidosis.

**Interventions:**

Corticosteroids, diuresis, guideline-directed medical therapy for heart failure.

**Outcomes:**

The patient’s symptoms and ventricular arrhythmias improved on steroids. Subsequent FDG-PET revealed increased uptake in a pattern consistent with CS.

**Conclusion:**

This clinical scenario highlights the importance of advanced cardiac imaging and clinical findings for the diagnosis of CS and exposes the practical need for a standardized, noninvasive strategy to the diagnosis of CS.

## 1. Introduction

Cardiac sarcoidosis (CS) is traditionally a histologic diagnosis. However, performing a myocardial biopsy comes with multiple limitations. The sensitivity of endomyocardial biopsy for CS is only around 20%–30%, even in patients with high clinical suspicion.^[1,2]^ This is a result of the patchy nature of cardiac involvement, with samples often showing fibrosis and lymphocytic infiltration without characteristic giant cells or epithelial granulomas.^[3]^ Furthermore, histopathologic features on their own are not enough to differentiate sarcoidosis from other granulomatous diseases. Histologic findings include granulomas, which involve concentric layers of immune cells with a center of macrophages and multinucleated giant cells, and an outer layer of sparse lymphocytes and occasional dendritic cells. However, these features can be shared by other clinical entities including tuberculosis, berylliosis, malignancies, autoimmune disorders, giant cell myocarditis, and other bacterial and fungal infections.^[3]^ As a result, the decision to start treatment for CS cannot depend exclusively on tissue. The advent of advanced imaging techniques has played an important role in the development of clinical criteria to diagnose or highly suspect CS in the absence of a tissue sample. This is a case where a non-invasive strategy led to the decision to initiate treatment for CS.

## 2. Case report

A 77-year-old male patient with a past medical history of heart failure with reduced ejection fraction (45%), coronary artery disease, lung cancer on remission with partial right lung resection, and chronic obstructive pulmonary disease presents after an episode of pressure-like, pleuritic, nonradiating substernal chest pain that lasted for 1 h. It was associated with shortness of breath, as well as a 1-week history of fatigue.

On admission, he was found to have bradycardia with 54 beats per minute, elevated blood pressure of 150/80 mmHg, and normal oxygen saturation on room air. Physical exam was notable for bibasilar lung crackles and bilateral pitting edema in lower extremities. Labs were notable for a mildly elevated troponin 0.034 ng/mL, elevated brain natriuretic peptide 504 pg/mL, normal creatinine 1.05 mg/dL, normal white blood cell count 8.8 × 10^3^μL, and normal hemoglobin 13.8 g/dL.

**Figure 1.**
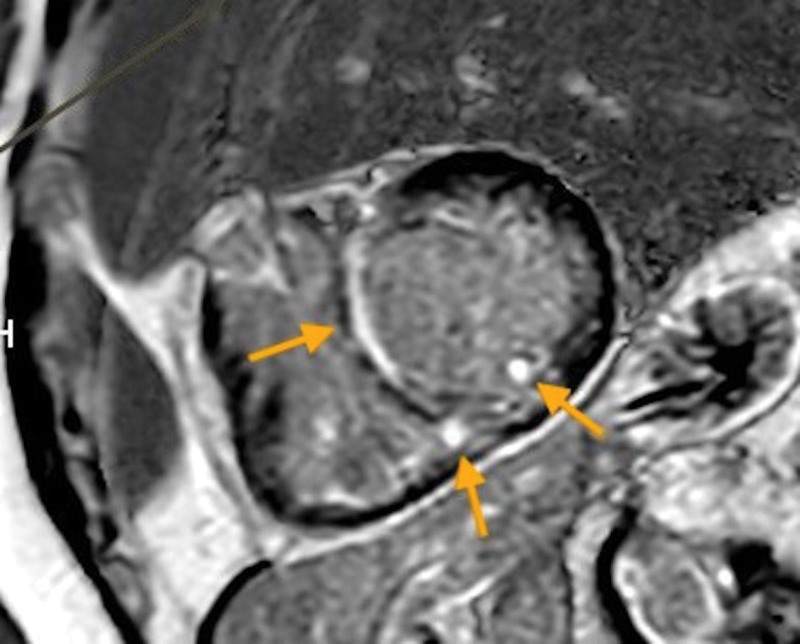
Cardiac magnetic resonance. Late gadolinium enhancement at the basal and midventricular septum, posterior right ventricular insertion, and papillary muscle (arrows). The subendocardium is spared.

Chest X-ray was unremarkable. Electrocardiogram was notable for first-degree atrioventricular block and multiple premature ventricular contractions (PVCs), which were present very frequently in telemetry monitoring. The echocardiogram was notable for a left ventricular ejection fraction (LVEF) of 35%, corresponding to a 10% drop from prior. It was also notable for left ventricle dilation and paradoxical septal motion. The patient underwent a coronary angiogram which revealed non-obstructive coronary artery disease. The patient subsequently underwent cardiac magnetic resonance (CMR) revealing late gadolinium enhancement at the basal midventricular septum, inferior wall, and posterior right ventricular insertion with spared subendocardium (Fig. 1). It also confirmed an LVEF of 34%.

The pattern of myocardial scarring on CMR raised suspicion for cardiac sarcoidosis. To treat cardiac rhythm abnormalities, the patient underwent PVC ablation with intracardiac echocardiography and electroanatomic mapping with pace mapping. There was successful ablation of one PVC morphology; however, the patient persisted with PVCs of different morphology, as well as runs of nonsustained ventricular tachycardia. The patient also persisted with symptoms of shortness of breath.

The patient subsequently received empiric treatment for sarcoidosis with oral steroids and amiodarone. The patient also received guideline-directed medical therapy (GDMT) for heart failure, including metoprolol, torsemide, and sacubitril/valsartan. Transjugular right ventricular endomyocardial biopsy was attempted. However, the procedure was aborted due to unfavorable anatomy. After a few days of treatment, there was improvement in PVCs and no further runs of non-sustained ventricular tachycardia. There was also improvement in shortness of breath.

**Figure 2.**
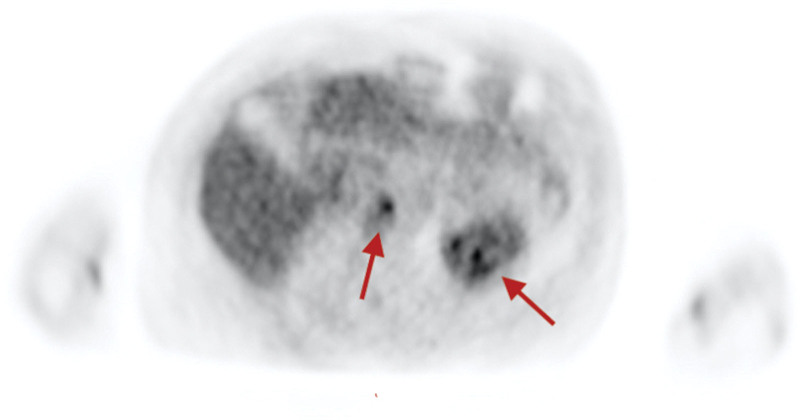
Cardiac magnetic resonance FDG-PET. Increased uptake in the cardiac segments predominantly seen in basal inferior and medial anteroseptal regions (arrows).

Subsequent CMR fluorodeoxyglucose-positron emission tomography (FDG-PET) revealed bilateral hilar uptake, as well as diffuse uptake in cardiac basal inferior, medial anteroseptal regions (Fig. 2), and papillary muscle. Placement of implantable cardioverter-defibrillator was planned as outpatient, and the patient was continued on oral steroids, amiodarone, and GDMT for heart failure.

## 3. Discussion

Diagnosis of CS can be challenging, especially considering that attempts to obtain histologic evidence can be of low yield, technically difficult, or impractical. Nevertheless, it can sometimes be crucial to promptly start treatment with corticosteroid or device implantation, even without biopsy results. As a result, advanced cardiac imaging, namely FDG-PET and CMR have become crucial for the diagnosis of CS.

FDG-PET detects areas of inflammation that results in pathologically increased glucose uptake, something characteristic of active CS.^[3]^ In a study comparing FDG-PET to posttransplant cardiac histologic assessment as gold standard, findings associated with high probability of CS had a sensitivity of 83.3% and a specificity of 100% for CS.^[4]^ Findings associated with high probability of CS included multiple, noncontiguous perfusion defects with associated FDG uptake as well as multiple areas of focal FDG uptake and extracardiac FDG uptake. In the same study, sensitivity was 100% and specificity 33% when the cutoff was lowered to any probable or highly probable finding. In another study comparing FDG-PET to the clinical diagnosis criteria of the Japanese Ministry of Health, Labor, and Welfare (MHLW) as gold standard, overall sensitivity of FDG-PET was 89% (95% CI, 79%–96%), and specificity 78% (95% CI, 68%–86%).^[5]^

CMR with late gadolinium enhancement (LGE) detects perfusion defects and fibrosis, which are characteristic of a later stage of CS.^[3]^ Findings that strongly suggest CS include intense LGE and prominent involvement of insertion points, along with contiguous and direct extension across the septum, with no alternative diagnosis.^[4]^ In a metanalysis that included 649 patients, CMR had an overall sensitivity of 0.93 (95% CI, 0.87–0.97) and specificity of 0.85 (95% CI, 0.68–0.94) in the diagnosis of CS.^[6]^ Another study of 58 patients that compared LGE-CMR with diagnostic criteria from the Japanese MHLW as gold standard, the sensitivity was 100% (95% CI, 78%–100%) and the specificity was 78% (95% CI, 64%–89%) for the diagnosis of CS.^[7]^

The Japanese Circulation Society (HCS) and the Heart and Rhythm Society (HRS) identified clinical findings that strongly suggest cardiac involvement of sarcoidosis (Table 1).^[3,8]^ These societies have established a “clinical diagnosis” group of CS, which defines criteria to confirm diagnosis of CS in patients with negative findings on myocardial biopsy or in whom biopsy was not performed.

**Table 1:** Clinical criteria suggestive of cardiac sarcoidosis.

Japanese Circulation Society 2016^[3]^	Heart Rhythm Society 2014^[8]^
*Major criteria*	1.Steroid +/− immunosuppressant responsive cardiomyopathy or heart block
1.High-grade atrioventricular block or fatal ventricular arrhythmias such as sustained ventricular tachycardia and ventricular fibrillation	2.Unexplained reduced VEGF (<40%)
2.LVEF <50% or focal ventricular wall asynergy	3.Unexplained sustained (spontaneous or induced) VT
3.Basal thinning of the ventricular septum or abnormal ventricular wall anatomy.	
4.^67^Ga citrate scintigraphy or ^18^F-FDG PET with abnormally high tracer accumulation in the heart.	4.Mobitz type II 2^nd^ degree heart block or 3rd-degree heart block
5.CMR with late contrast enhancement of the myocardium.^[1]^	5.Patchy uptake on dedicated cardiac PET (in a pattern consistent with CS)
*Minor criteria*	
1.Ventricular arrhythmias (NSVT, multifocal of frequent PVCs), bundle branch block, axis deviation, or abnormal Q waves	6.Late Gadolinium Enhancement on CMR (in a pattern consistent with CS) Positive gallium uptake (in a pattern consistent with CS)
2.Perfusion defects on myocardial perfusion scintigraphy	
Endomyocardial biopsy with monocyte infiltration and moderate or severe interstitial fibrosis	

CMR = cardiac magnetic resonance, CS = cardiac sarcoidosis, FDG PET = fluorodeoxyglucose-positron emission tomography, LVEF = left ventricular ejection fraction, NSVT = nonsustained ventricular tachycardia, PVCs = premature ventricular contractions, VT = ventricular tachycardia.

Interestingly, the JCS established one set of clinical criteria that do not require extracardiac histologic diagnosis. It involves a combination of cardiac and pulmonary or ophthalmic clinical findings that strongly suggest sarcoidosis, together with highly suggestive laboratory findings. However, the diagnosis CS without histologic evidence came into question in a study that found low concordance between JCS and other major diagnostic tools for CS.^[9]^

There is great need for more research on new modalities for the effective and practical diagnosis of CS, regardless of the involvement of an invasive procedure. Performing simultaneous FDG-PET and CMR to diagnose CS is promising, as it exploits the key advantages of each imaging modality.^[10]^ Fusion of different types of imaging can provide additional information, such as by studying the relationship between increased tracer uptake and fibrosis. Endomyocardial biopsy assisted by cardiac imaging including CMR and PET had an increase in sensitivity from 32% to 55% in a study on patients who had a prior biopsy without the assistance of imaging.^[11]^ Magnetic resonance imaging with T2 mapping also shows promise and is undergoing investigation.^[12]^

New techniques of robotic-assisted myocardial biopsy guided by electroanatomic mapping, which involves the integration of multiple imaging modalities, have the potential for increased accuracy and lower complication rates.^[13]^

## Author contributions

Conceptualization: Jose Aguilar-Gallardo

Investigation: Jose Aguilar-Gallardo, Javier Arreaza, Johanna Contreras

Methodology: Alaa Omar, Javier Arreaza

Writing – original draft: Jose Aguilar-Gallardo

Writing – review & editing: Glenmore Lasam, Alaa Omar, Johanna Contreras

## References

[1] Sekiguchi M Numao Y Imai M Furuie T Mikami R. Clinical and histopathological profile of sarcoidosis of the heart and acute idiopathic myocarditis concepts through a study employing endomyocardial biopsy I. Sarcoidosis: symposium on secondary myocardial disease. Jpn Circ J. 1980;44:249–63. 7373823 10.1253/jcj.44.249

[2] Uemura A Morimoto S-i Hiramitsu S Kato Y Ito T Hishida H. Histologic diagnostic rate of cardiac sarcoidosis: evaluation of endomyocardial biopsies. Am Heart J. 1999;138:299–302. 10426842 10.1016/s0002-8703(99)70115-8

[3] Terasaki F Azuma A Anzai T. JCS 2016 guideline on diagnosis and treatment of cardiac sarcoidosis ― digest version ―. Circ J. 2019;83:2329–88. 31597819 10.1253/circj.CJ-19-0508

[4] Divakaran S Stewart GC Lakdawala NK Padera RF Zhou W Desai AS. Diagnostic accuracy of advanced imaging in cardiac sarcoidosis. Circ Cardiovasc Imaging. 2019;12:e008975. 31177817 10.1161/CIRCIMAGING.118.008975 PMC6653689

[5] Youssef G Leung E Mylonas I Nery P Williams K Wisenberg G. The use of 18F-FDG PET in the diagnosis of cardiac sarcoidosis: a systematic review and metaanalysis including the Ontario experience. J Nucl Med. 2012;53:241–8. 22228794 10.2967/jnumed.111.090662

[6] Zhang J Li Y Xu Q Xu B Wang H. Cardiac magnetic resonance imaging for diagnosis of cardiac sarcoidosis: a meta-analysis. Can Respir J. 2018;2018:7457369–7457369. 30651895 10.1155/2018/7457369 PMC6311842

[7] Smedema J-P Snoep G van Kroonenburgh MPG. Evaluation of the accuracy of gadolinium-enhanced cardiovascular magnetic resonance in the diagnosis of cardiac sarcoidosis. J Am Coll Cardiol. 2005;45:1683–90. 15893188 10.1016/j.jacc.2005.01.047

[8] Birnie DH Sauer WH Bogun F Cooper JM Culver DA Duvernoy CS. HRS expert consensus statement on the diagnosis and management of arrhythmias associated with cardiac sarcoidosis. Heart Rhythm. 2014;11:1304–1323. 10.1016/j.hrthm.2014.03.043 24819193

[9] Ribeiro Neto ML Jellis C Hachamovitch R Wimer A Highland KB Sahoo D. Performance of diagnostic criteria in patients clinically judged to have cardiac sarcoidosis: Is it time to regroup? Am Heart J. 2020;223:106–109. 32240829 10.1016/j.ahj.2020.02.008

[10] White JA Rajchl M Butler J Thompson RT Prato FS Wisenberg G. Active cardiac sarcoidosis. Circulation. 2013;127:e639–41. 23733970 10.1161/CIRCULATIONAHA.112.001217

[11] Kandolin R Lehtonen J Graner M. Diagnosing isolated cardiac sarcoidosis. J Intern Med. 2011;270:461–8. 21535250 10.1111/j.1365-2796.2011.02396.x

[12] Trivieri MG Robson P LaRocca G. Abstract 16511: hybrid magnetic resonance imaging and fluorodeoxyglucose (FDG) positron emission tomography (PET) guided cardiac sarcoidosis treatment. Circulation. 2018;138(Suppl_1):A16511–A16511.

[13] Bhimaraj A Trachtenberg B Valderrábano M. Robotically guided left ventricular biopsy to diagnose cardiac sarcoidosis. Circ Heart Fail. 2018;11:e004627. 29519901 10.1161/CIRCHEARTFAILURE.117.004627

